# Integrated analysis of fine-needle-aspiration cystic fluid proteome, cancer cell secretome, and public transcriptome datasets for papillary thyroid cancer biomarker discovery

**DOI:** 10.18632/oncotarget.23951

**Published:** 2018-01-04

**Authors:** Chia-Chun Wu, Jen-Der Lin, Jeng-Ting Chen, Chih-Min Chang, Hsiao-Fen Weng, Chuen Hsueh, Hui-Ping Chien, Jau-Song Yu

**Affiliations:** ^1^ Graduate Institute of Biomedical Sciences, Chang Gung University, Taoyuan, Taiwan; ^2^ Department of Cell and Molecular Biology, College of Medicine, Chang Gung University, Taoyuan, Taiwan; ^3^ Molecular Medicine Research Center, Chang Gung University, Taoyuan, Taiwan; ^4^ Division of Endocrinology and Metabolism, Department of Internal Medicine, Chang Gung Memorial Hospital at Linkou, Taoyuan, Taiwan; ^5^ Department of Pathology, Chang Gung Memorial Hospital at Linkou, Taoyuan, Taiwan; ^6^ Liver Research Center, Chang Gung Memorial Hospital at Linkou, Taoyuan, Taiwan; ^7^ Department of Surgery, Department of Medical Research and Development Linkou Branch, Chang Gung Memorial Hospital at Linkou, Taoyuan, Taiwan

**Keywords:** papillary thyroid carcinoma, fine needle aspiration cystic fluid, proteome profiles, secretome, biomarker

## Abstract

Thyroid ultrasound and ultrasound-guided fine-needle aspiration (USG/FNA) biopsy are currently used for diagnosing papillary thyroid carcinoma (PTC), but their detection limit could be improved by combining other biomarkers. To discover novel PTC biomarkers, we herein applied a GeLC-MS/MS strategy to analyze the proteome profiles of serum-abundant-protein-depleted FNA cystic fluid from benign and PTC patients, as well as two PTC cell line secretomes. From them, we identified 346, 488, and 2105 proteins, respectively. Comparative analysis revealed that 191 proteins were detected in the PTC but not the benign cystic fluid samples, and thus may represent potential PTC biomarkers. Among these proteins, 101 were detected in the PTC cell line secretomes, and seven of them (NPC2, CTSC, AGRN, GPNMB, DPP4, ERAP2, and SH3BGRL3) were reported in public PTC transcriptome datasets as having 4681 elevated mRNA expression in PTC. Immunoblot analysis confirmed the elevated expression levels of five proteins (NPC2, CTSC, GPNMB, DPP4, and ERAP2) in PTC versus benign cystic fluids. Immunohistochemical studies from near 100 pairs of PTC tissue and their adjacent non-tumor counterparts further showed that AGRN (*n =* 98), CTSC (*n =* 99), ERAP2 (*n =* 98) and GPNMB (*n =* 100) were significantly (*p* < 0.05) overexpressed in PTC and higher expression levels of AGRN and CTSC were also significantly associated with metastasis and poor prognosis of PTC patients. Collectively, our results indicate that an integrated analysis of FNA cystic fluid proteome, cancer cell secretome and tissue transcriptome datasets represents a useful strategy for efficiently discovering novel PTC biomarker candidates.

## INTRODUCTION

Thyroid cancer is the most prevalent malignant endocrine carcinoma in the world. In America, the incidence rate of thyroid cancer increased an average of 4.6% per year between 2004 and 2013, and the disease is now ranked as the third most common cancer in women [1]. Among all of the histological types of thyroid cancer, papillary thyroid carcinoma (PTC) accounts for the majority (80–85%) of cases. Most PTC patients have a good prognosis with a 10-year survival rate > 90%; however, a small portion of PTCs are aggressive and may develop distant metastases that are associated with higher mortality [2]. Currently, preoperative ultrasound-guided fine-needle aspiration (USG/FNA) followed by cytopathologic diagnosis is the standard procedure for examining thyroid nodules and determining therapeutic modalities [3]. Among all cases, however, 10–20% yield indeterminate biopsy results on preoperative USG/FNA diagnosis. These patients normally undergo thyroidectomy, but this is often unnecessary, as post-surgery immunohistological analysis has shown that 80% of the suspicious cases are benign [4, 5]. Thus, if we hope to reduce unnecessary thyroidectomies and prevent deterioration in PTC, we urgently need reliable biomarkers that can distinguish between benign and malignant nodules in patients with indeterminate lesions.

Cystic fluid and tissue/cell specimens obtained from thyroid nodules during the FNA procedure represent ideal resources for discovering and verifying PTC biomarkers. Various types of potential PTC biomarkers have been explored in FNA cystic fluid and tissue/cell specimens, including DNA mutations/rearrangements and proteins. Several gene mutations have been identified in thyroid cancer. For example, somatic *RET/PTC* gene rearrangements and the *BRAF*^V600E^ point mutation are frequently found in PTC, and clinicians have used them to decide whether or not to undertake thyroidectomy [2, 6]. However, FNA cystic fluid samples typically contain very few cancer cells, limiting their usefulness for cytologic evaluation or genetic marker screening, and creating ambiguity in the diagnosis of thyroid cancer [7].

To address this issue, several groups have applied proteomic approaches to discover potential PTC biomarkers from FNA cystic fluid samples, PTC cell secretomes, or tissue specimens. For example, two studies identified secreted proteins (secretome analysis; 154 and 83 proteins, respectively) from PTC cell lines using LC-MS/MS analysis [8, 9]. Two other studies investigated the differential protein profiles between benign and malignant cystic fluids using two-dimensional gel electrophoresis/MALDI-TOF-MS or iTRAQ-based LC-MS/MS. These studies then further verified two and three proteins, respectively, by Western blotting, ELISA and/or immunohistochemical analysis of PTC cystic fluids and tissue samples [10, 11]. More recently, Martínez-Aguilar et al. used MS-based quantitative expression analysis of over 1600 proteins in normal thyroid tissues versus PTC to identify ∼180 proteins that are deregulated in PTC tumors [12]. Although these proteomic studies have discovered numerous proteins as potential PTC biomarkers, however, challenges remain in determining how researchers should efficiently prioritize and select targets for further validation in a large PTC sample cohort.

In this study, we used an integrated omics approach to identify potential PTC biomarkers. We applied a GeLC-MS/MS strategy to comprehensively analyze the proteome profiles of abundant-protein-depleted FNA cystic fluids (i.e., those depleted of the top 14 most abundant proteins) from benign and PTC patients, as well as secretomes from the PTC cell lines, BHP 7-13 and CGTH W3. Using a criterion of at least two peptide hits for confident protein identification, we identified 346 and 488 proteins expressed in the FNA cystic fluids of benign and PTC patients, and 2105 proteins in the secretomes of the two PTC cell lines. Integrated analysis of these three datasets revealed that 101 proteins found in the PTC cystic fluid but not the corresponding benign samples were also detected in the PTC cell line secretome. We then combined two publicly available mRNA microarray datasets representing PTC tissues, and used them to identify the seven strongest candidates: NPC2, CTSC, AGRN, GPNMB, DPP4, ERAP2, and SH3BGRL3. Finally, we used Western blot analysis and/or immunohistochemistry to confirm the up-regulations of selected candidates in PTC specimens, and evaluated their associations with the clinicopathological characteristics of PTC patients.

## RESULTS

### Generating a thyroid cystic fluid proteome dataset using GeLC-MS/MS

**Figure 1:**
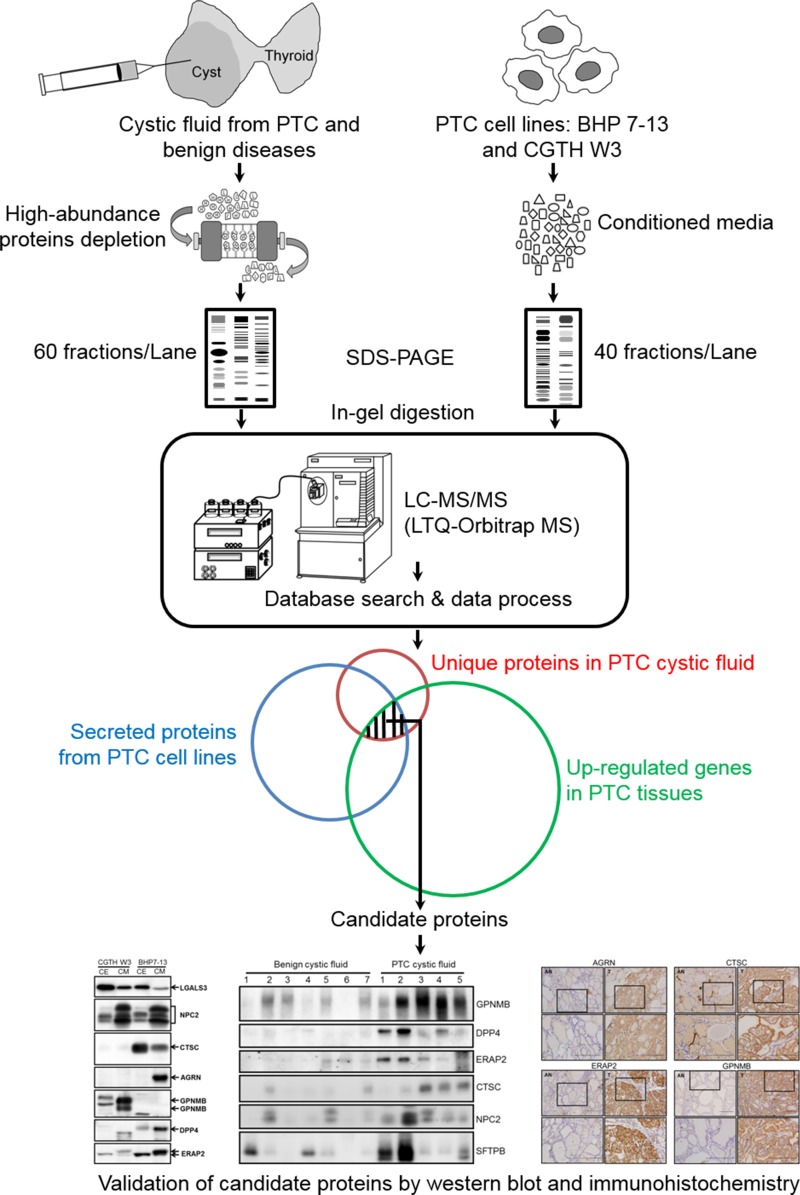
Strategy for identifying potential PTC biomarkers Schematic representation of the experimental design used in this study. We first applied a GeLC-MS/MS approach to comprehensively analyze the proteome profiles of thyroid cystic fluid samples from PTC and benign lesions, as well as the secretome profiles of two PTC cell lines. Meanwhile, we searched public-domain transcriptome datasets for genes whose transcriptional levels were up-regulated in PTC tissues. We then integrated the three datasets to identify candidate genes/proteins that were selectively detected in the PTC cystic fluid samples, highly up-regulated in PTC tissues, and secreted/released from PTC cells. Finally, we verified the candidate proteins in FNA cystic fluid and PTC tissue samples using immunoblotting and immunohistochemistry.

**Figure 2:**
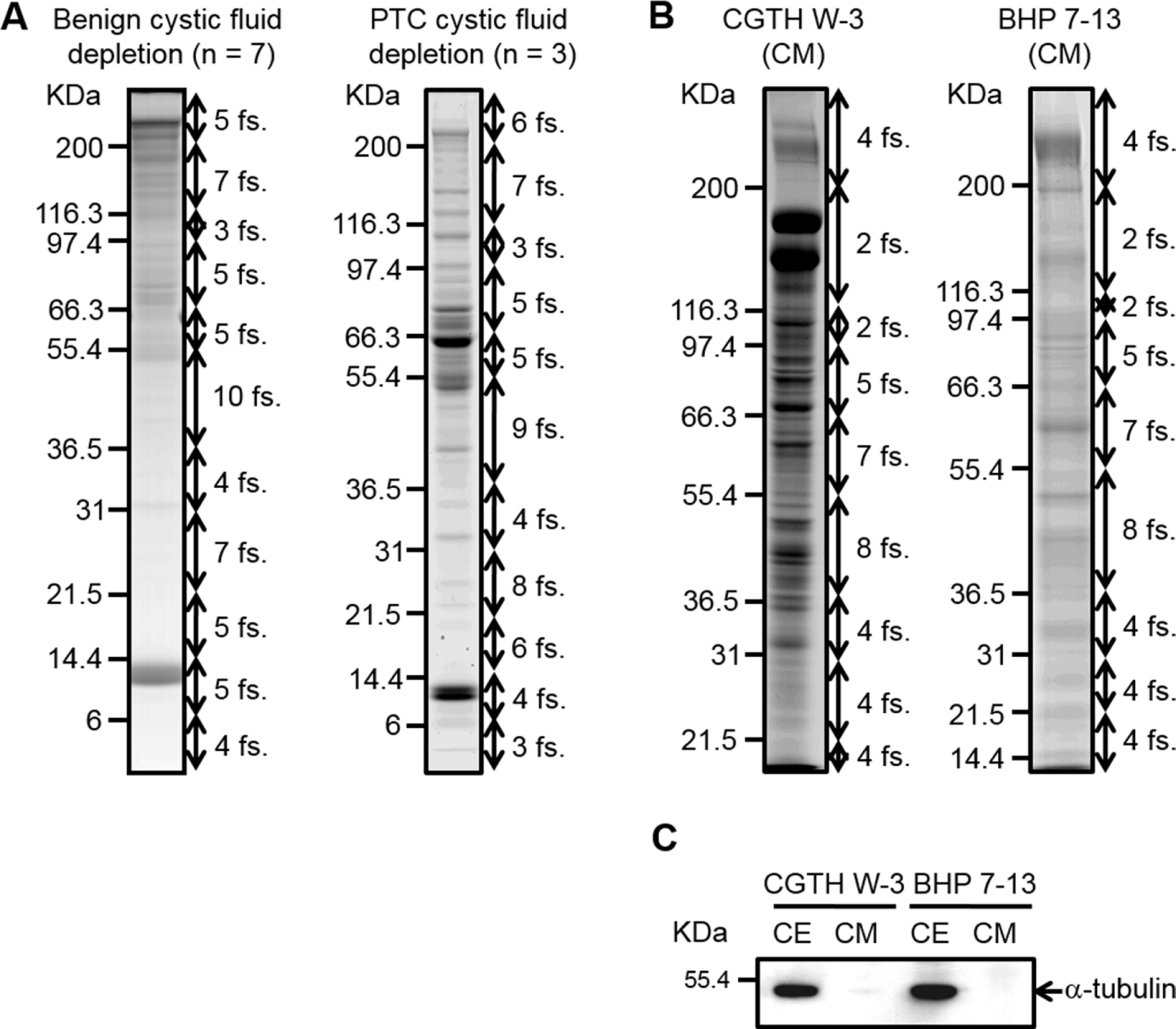
SDS-PAGE analysis of thyroid cystic fluid samples and conditioned media harvested from PTC cells (**A**) Thyroid cystic fluid samples of seven benign cases and three PTC cases were independently pooled and depleted using Hu-14 columns. The depleted proteins (60 μg) were resolved on 8–14%-gradient SDS gels and stained with Coomassie Blue. The gel lanes were sliced into 60 fractions (*fs.*) for further analysis. (**B**) The conditioned media (*CM*) of CGTH W3 and BHP 7-13 cells were collected and processed as described in the “Experimental Procedures.” Proteins (50 μg) from the concentrated CM were resolved on 8–14%-gradient SDS gels and stained with Coomassie Blue. The gel lanes were sliced into 40 pieces for further analysis. (**C**) Proteins (40 μg) from the CM and extracts of PTC cell lines (*CE*) were resolved by SDS-PAGE and subjected to Western blot analysis using an antibody against α-tubulin.

The strategy we used to improve the identification of novel PTC biomarkers is delineated in Figure 1. We used USG/FNA to collect thyroid cystic fluid samples from 12 patients: five cases of PTC and seven cases of benign disease (Supplementary Table 1). The individual cystic fluid samples from seven benign and three PTC specimens were pooled into two groups containing equal amounts of protein. These samples were subjected to top-14-high-abundance-protein depletion followed by GeLC-MS/MS-based proteomic profiling. The protein staining patterns of the abundant-protein-depleted fractions are shown in Figure 2A, and were quite different from those of the un-depleted (crude) and abundant protein fractions (Supplementary Figure 1). Our results demonstrated that the highly abundant proteins accounted for >90% of the total protein mass in the thyroid cystic fluid, as previously seen in human serum [13, 14]. The gel lane of each sample was sliced into 60 fractions, and the proteins were subjected to in-gel tryptic digestion and identified by LC-MS/MS analysis using an LTQ-Orbitrap. Supplementary Table 2A lists the MS-identified proteins in the abundant protein fractions of benign cystic fluid, as captured by a Human 14 MARS column. To examine if the combined use of these technology platforms (i.e., abundant protein depletion and GeLC-MS/MS) can significantly improve the detection rate of low-abundance proteins in the thyroid cystic fluid samples, we used the same GeLC-MS/MS approach to analyze respectively the undepleted and abundant protein-depleted cystic fluid sample pooled from three PTC specimens. This analysis identified 241 proteins in the undepleted sample (Supplementary Table 2B), which is much less than the number of proteins (488) identified in the depleted sample (Supplementary Table 3B). Regarding the total identified spectra, there was a high proportion (45%, 24689/54411) of abundant plasma proteins in the undepleted sample as compared to a significantly lower proportion (19%, 22474/115905) of these abundant plasma proteins in the depleted sample (Supplementary Table 2C). After removing the abundant plasma protein identities from these two datasets, 194 proteins and 456 proteins remained respectively in the undepleted and depleted sample; importantly, we found that 284 proteins could only be identified in the depleted sample. Collectively, these findings confirmed that abundant plasma protein depletion is very useful to significantly increase the detection rate of low-abundance proteins in thyroid cystic fluid.

From the benign and PTC abundant-protein-depleted samples (60 μg each), the GeLC-MS/MS approach identified 346 and 488 proteins, respectively, with multiple (≧2) peptide hits and a false discovery rate (FDR) of 0.58–1.23% (Table 1 and Supplementary Table 3A–3B). Recent studies have reported galectin-3 (LGALS3) as a reliable immunohistochemical and serum biomarker for PTC detection [15, 16]. Thus, it is notable that we identified galectin-3 in our FNA cystic fluid dataset, and our label-free spectral counting approach found that its expression level was much higher (∼7.8 fold) in our PTC samples compared to our benign cystic fluid samples (Supplementary Table 4).

**Table 1:** Number of proteins identified in thyroid cystic fluids and PTC cell secretomes

Thyroid cystic fluid or PTC cell lines	No. of identified proteins^a^	FDR^b^
Benign cystic fluid	346	0.58%
PTC cystic fluid	488	1.23%
BHP 7-13	830	0.12%
CGTH W-3	1921	0.21%

^a^The numbers represent identified proteins for which at least two unique peptides of 95% peptide possibility and 95% protein possibility were identified using the Scaffold software.

^b^The false discovery rate (FDR) was calculated as the ratio of the spectra assigned to a random database over those assigned to a normal database.

### GeLC-MS/MS-based secretomic analysis of two PTC cell lines

Serum-free conditioned media of two PTC cell lines (CGTH W3 and BHP 7-13) were concentrated and desalted, and 50 μg of proteins from each sample were resolved by 8–14% gradient SDS-PAGE (Figure 1). The protein staining patterns of the conditioned media from CGTH W3 and BHP 7-13 cells are shown in Figure 2B. As a quality control, we used Western blotting analysis to examine the distribution of α-tubulin, a cytoplasmic abundant protein, between total cell extracts and the conditioned media. We clearly detected α-tubulin in the total cell extracts but obtained little or no such signal from the conditioned media (Figure 2C), confirming that the secreted/shed proteins had been specifically collected from the cultured PTC cell lines. The gel lane of each sample was sliced into 40 fractions, and the proteins were identified by the above-described GeLC-MS/MS approach. Starting from 50 μg of proteins from the conditioned media, we identified 1921 and 830 proteins in the CGTH W3 and BHP 7-13 cell lines, respectively, with a FDR of 0.12–0.21% (Table 1 and Supplementary Table 3C–D). Our analysis thus yielded a total of 2105 proteins that served as our dataset for PTC biomarker selection (Supplementary Table 5A).

### Integrated analysis and secretion-pathway prediction of the MS-identified proteins in the thyroid cystic fluid and PTC cell secretome

**Figure 3:**
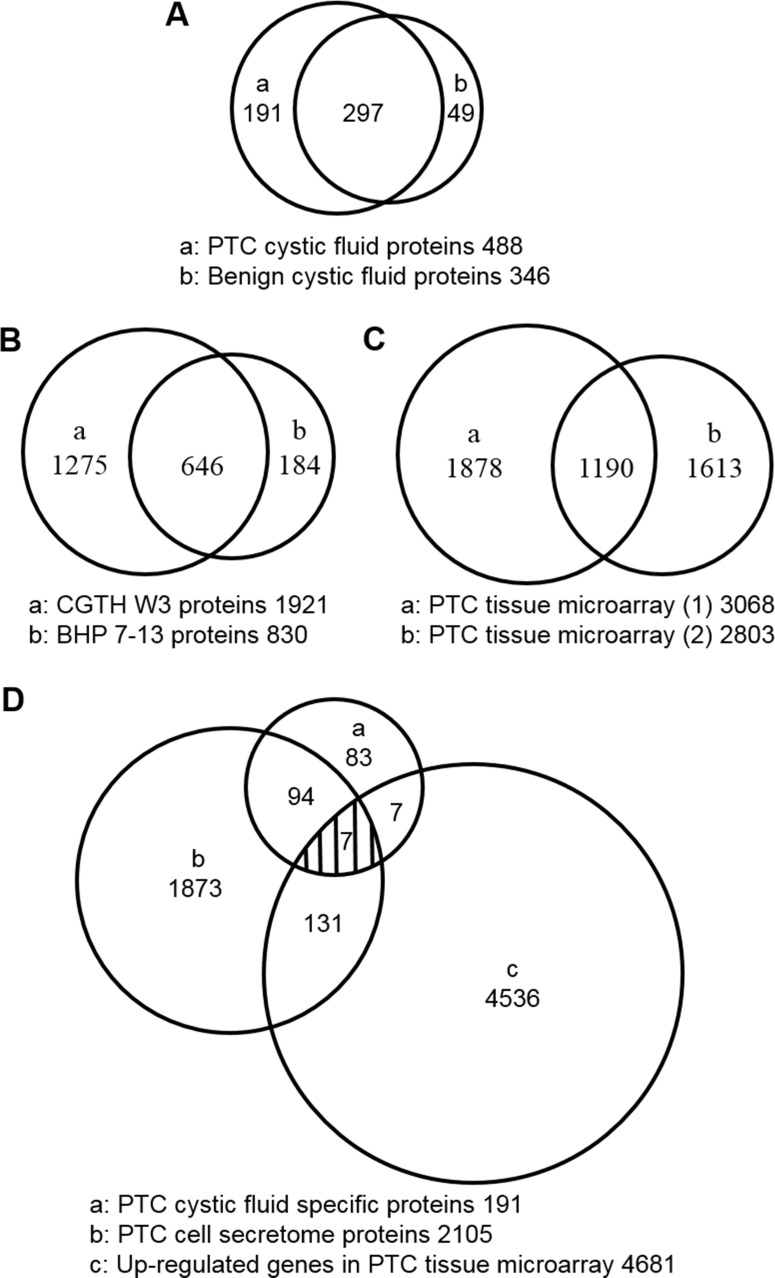
Venn diagrams showing the overlap of proteins in the thyroid cystic fluid, PTC cell secretome, and PTC tissue microarray datasets (**A**) The number of proteins identified in thyroid cystic fluid samples from PTC patients and patients with benign lesions. (**B**) The numbers of proteins identified in the secretomes of the PTC cell lines, CGTH W3 and BHP 7-13. (**C**) The numbers of up-regulated genes in the two PTC tissue transcriptome datasets. (**D**) Integrated analysis of the PTC cystic fluid proteome (191 proteins), the PTC cell secretome dataset (2105 proteins), and the up-regulated genes in the two PTC tissue transcriptome datasets (4681 genes). This analysis identified seven proteins as strong PTC biomarker candidates.

A total of 537 unique proteins were detected in the benign and PTC cystic fluid proteomes; of them, 297 were shared between the datasets (Figure 3A and Supplementary Table 4). Using a label-free quantification strategy and spectral counting, we determined that 86 of the shared proteins (29%) were up-regulated and 36 (12%, 36 of 297) were down-regulated more than two fold in the PTC versus benign samples (Supplementary Table 4). The up-regulated proteins included some potential biomarkers previously reported as having elevated levels in PTC [10, 15–28], such as FTL, FTH1, LGALS3, JUP, S100A11, SERPINA1, TF, ECM1, CD163, BCHE, SOD2, CP, FN1, S100A4, and C5 (Supplementary Table 4). This confirms that our label-free quantification strategy can be used to discover PTC biomarkers from proteomic datasets generated by abundant protein depletion of cystic fluid samples coupled with GeLC-MS/MS.

The identified proteins were further analyzed using bioinformatics programs designed to predict protein secretion pathways. Among the 537 non-redundant proteins identified in our thyroid cystic fluid samples, 246 proteins were predicted to be classical secreted proteins, as assessed by the SignalP 4.0 program (SignalP-TM probability ≧0.50, SignalP-noTM ≧0.45) based on the presence of a signal peptide in target proteins with or without transmembrane (TM) sequences [29]. The SecretomeP 2.0 program predicted that 131 proteins would be released through the non-classical pathway (SignalP score **<** 0.5 or 0.45 and Secretome score ≧0.50) [30]. Among them, only five proteins were determined to be integral membrane proteins, as assessed by TMHMM [31]. Among the 2105 proteins of the PTC cell secretome dataset, 426 were predicted to be classical secreted proteins, 723 were predicted to be non-classical secreted proteins, and 81 were predicted to be integral membrane proteins (Table 2). Taken together, our results indicated that 71.1% (382 of 537) of the cystic fluid proteins and 58.4% (1230 of 2105) of the conditioned-media proteins of cultured PTC cells could be secreted/released via different mechanisms. Notably, the cystic fluid proteins of the up-regulated group described above included a higher percentage (84.9%, 73 of 86) of secretory proteins than those of the down-regulated group (63.9%, 23 of 36) (Supplementary Tables 4 and 6). Moreover, among the 537 cystic fluid proteins, the 191 PTC-unique proteins included a relatively high percentage (74.3%, 142 of 191) of secretory proteins (Table 2, Supplementary Table 4 and Supplementary Table 6) compared to those uniquely identified in the benign cystic fluid sample (65.3%, 32 of 49) (Supplementary Tables 4 and 6). These results indicate that most PTC-related proteins are predicted to be secretory, and thus PTC biomarkers could be exploited in cystic fluid. In addition, we used the Gene Ontology (GO) annotation tool in the DAVID Bioinformatics Resources (v6.8) to perform functional annotation of those 277 proteins with two-fold up-regulation or solely detected in PTC cystic fluids, including biological process, molecular function and proteins location of cellular components. Regarding the biological process, the top three categories were platelet degranulation, complement activation (classical pathway) and complement activation (Supplementary Figure 2A). The major molecular functions were classified as serine-type endopeptidase activity, structural molecule activity and heparin binding (Supplementary Figure 2B). The protein localization was mainly classified into extracellular such as extracellular exosome, extracellular space and extracellular region (Supplementary Figure 2C).

**Table 2:** Predicted secretion pathways of proteins identified in thyroid cystic fluids and the two PTC cell secretomes

Thyroid cystic fluid and PTC cell lines	No. of identified proteins				Percentage of predicted secreted proteins (%)
	Classical secretion^*b*^	Nonclassical secretion^*c*^	Membrane protein^*d*^	Others^*e*^	
Benigh cystic fluid	163	71	5	107	69.1
PTC cystic fluid	232	115	3	138	71.7
Benign U PTC cystic fluid	246	131	5	155	71.1
PTC cystic fluid alone^*a*^	83	59	0	49	74.3
CGTH W3	321	683	73	844	56.1
BHP 7-13	290	209	22	309	62.8
CGTH W3 U BHP 7-13	426	723	81	875	58.4

^a^A total of 191 proteins were identified as being unique to PTC from the thyroid cystic fluid dataset.

^b^Proteins predicted by the SignalP program to be secreted via the classical secretion pathway (SignalP probability ≥ 0.45 without TM; ≥ 0.50 with TM).

^c^Proteins predicted to be secreted by the nonclassical secretion pathway, as assessed using SignalP and SecretomeP (SignalP probability lower than the above criteria and SecretomeP score ≥ 0.50).

^d^Proteins predicted by the TMHMM to form integral membrane proteins that were not predicted to be secreted via the classical or nonclassical secretion pathways.

^e^Proteins that could not be classified as classical secreted, nonclassical secreted, or integral membrane proteins.

### Selecting novel PTC cystic fluid biomarker candidates through a combined analysis of the PTC cystic fluid proteome, cell secretome, and tissue transcriptome

To search for PTC-specific biomarkers, the 191 proteins (Figure 3A) uniquely detected in PTC cystic fluid samples were compared with the 2105 proteins (Figure 3B) identified in the secretomes of the BHP 7-13 and CGTH W3 cell lines. We also mined two public cDNA microarray datasets (the E-GEOD-3678 dataset from EBI-ArrayExpress and the GDS1665 dataset from Gene Expression Omnibus) for a total of 4681 genes whose tissue mRNA expression levels were reported to be significantly elevated in PTC versus adjacent normal tissues (Figure 3C and Supplementary Table 7) [32]. Our combined analysis of the three datasets (Figure 3D) identified seven highly relevant potential PTC candidate biomarkers: epididymal secretory protein E1 (NPC2), dipeptidyl peptidase 1 (CTSC), agrin (AGRN), transmembrane glycoprotein NMB (GPNMB), dipeptidyl peptidase 4 (DPP4), endoplasmic reticulum aminopeptidase 2 (ERAP2), and SH3 domain-binding glutamic acid-rich-like protein 3 (SH3BGRL3) (Table 3). All seven are predicted to be secreted via the classical or nonclassical secretion pathways, and four of them (CTSC, AGRN, DPP4, and SH3BGRL3) are known as exosomal proteins [33]. Of them, DPP4 was previously detected in the human serum proteome dataset [34] (Table 3), and was verified as a potential PTC biomarker using tissue specimens [35]. Otherwise, ERAP2 was also elucidated highly expressed in PTC tissue with cervical lymph node metastasis [36], but other five candidates had not previously been reported.

**Table 3:** List of seven high-potential candidate proteins for PTC biomarkers

Swiss-Prot		Gene name	Protein Name	Classical secretion	Nonclassical secretion	Membrane protein	HPPP^*a*^	ExoCarta^*b*^
ID_HUMAN	Code							
NPC2^c^	P61916	*NPC2*	Epididymal secretory protein E1	V				
CATC	P53634	*CTSC*	Dipeptidyl peptidase 1	V				V
AGRIN^c^	O00468	*AGRN*	Agrin	V				V
GPNMB	Q14956	*GPNMB*	Transmembrane glycoprotein NMB	V		V		
DPP4^c^	P27487	*DPP4*	Dipeptidyl peptidase 4	V		V	V	V
ERAP2	Q6P179	*ERAP2*	Endoplasmic reticulum aminopeptidase 2		V			
SH3L3	Q9H299	*SH3BGRL3*	SH3 domain-binding glutamic acid-rich-like protein 3		V			V

^a^Proteins were detected in the Human Plasma Proteome Project (HPPP) database, which contains 3020 proteins.

^b^Proteins were detected in the human exosome protein database of ExoCarta.

^c^The proteins were previously reported as potential PTC biomarkers.

### Verifying potential PTC biomarkers in the conditioned media of PTC cell lines and thyroid cystic fluids

**Figure 4:**
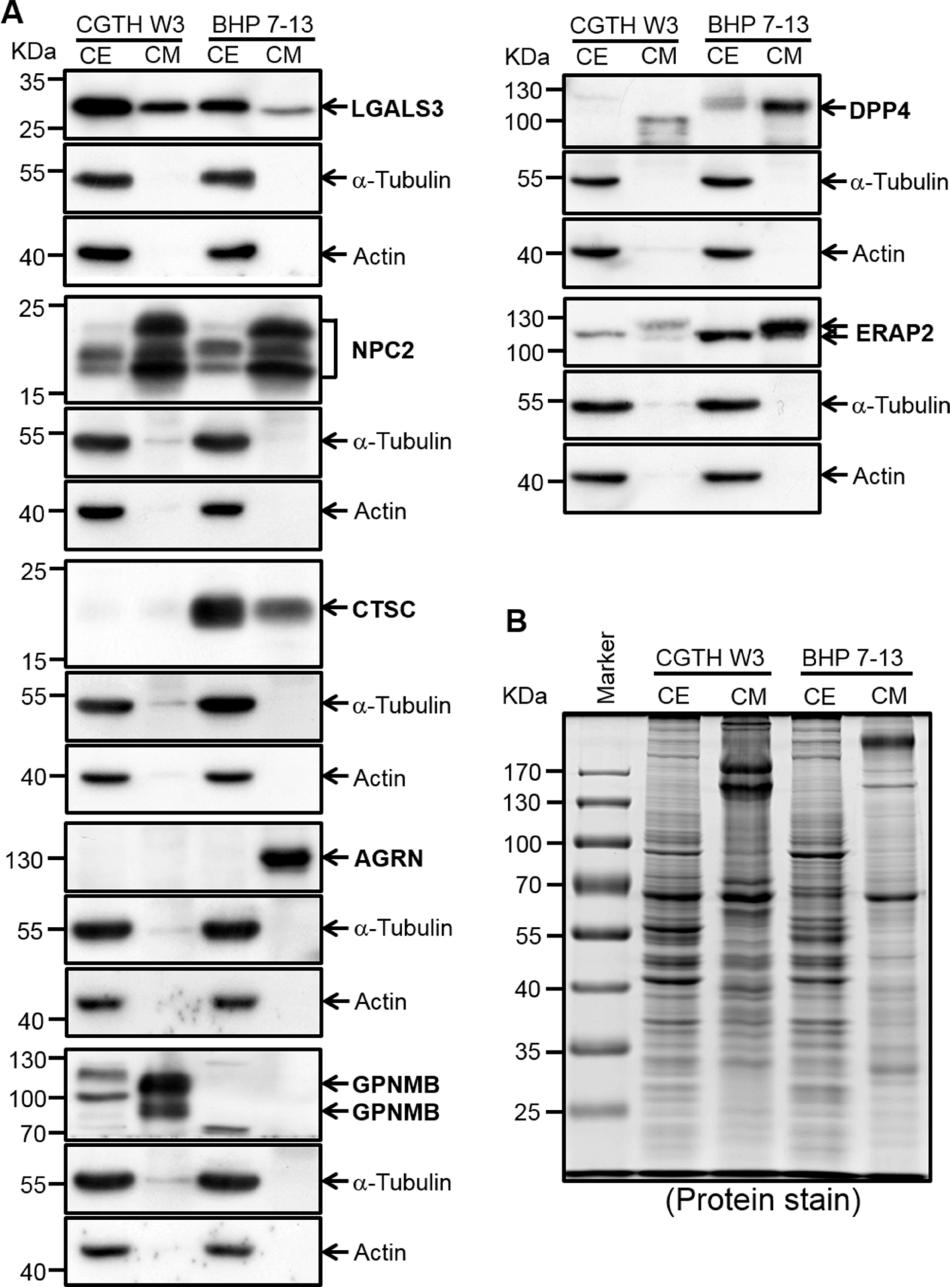
Verification of potential PTC biomarkers in PTC cell extracts and conditioned media by Western blot analysis (**A**) Proteins (50 μg) from conditioned media (*CM*) and cell extracts (*CE*) were subjected to Western blot analysis. The utilized PTC cell lines are denoted at the top of the blot. LGALS3, which was previously reported as a potential PTC biomarker, was also included in this analysis. Two cytosolic proteins, α-tubulin and actin, were detected as controls. (**B**) The Coomassie Blue-stained protein profile was used as a loading control.

**Figure 5:**
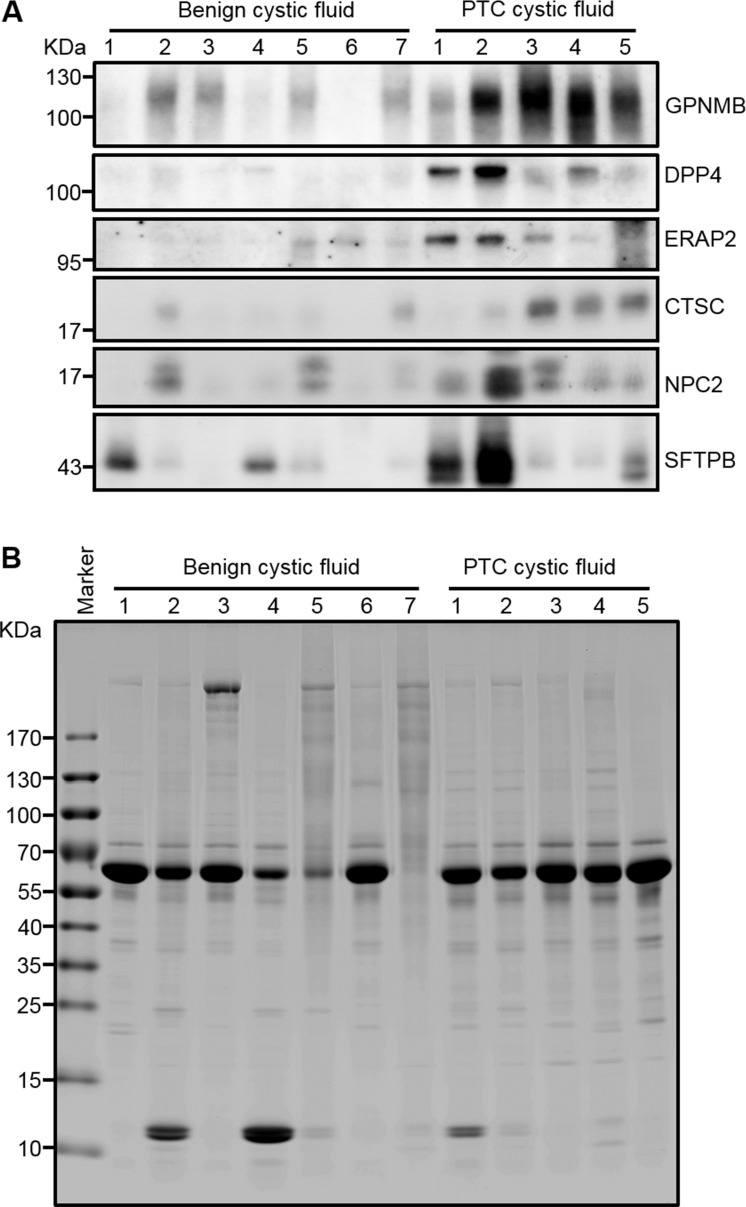
Verification of GPNMB, ERAP2, CTSC, and NPC2 in individual FNA cystic fluid samples by Western blot analysis (**A**) Equal amounts of proteins (20 μg) prepared from individual FNA cystic fluid samples were separated by 6-18%-gradient SDS-PAGE, transferred to PVDF membranes, and probed with specific antibodies against the indicated target proteins. SFTPB, which was previously reported as a potential PTC biomarker, was also included in this analysis. (**B**) The Coomassie-Blue-stained protein profile was used as a loading control.

We used Western blot analysis to verify the seven selected biomarkers in conditioned media of the CGTH W3 and BHP 7-13 cell lines, as well as in cell extracts. Galectin-3, which was previously reported as a promising PTC biomarker [37], was used as a positive control. As shown in Figure 4, all of the tested proteins except SH3BGRL3 could be detected in the conditioned media of CGTH W3 and/or BHP 7-13 cells, and their relative expression levels fitted well with the total spectral numbers detected in the PTC cell secretome (Supplementary Table 5B). Similar experiments were performed to evaluate the levels of these potential biomarkers in twelve abundant-protein-depleted cystic fluid samples including ten samples used for initial discovery experiment and two subsequently collected PTC cystic fluid samples. As shown in Figure 5A, five candidates (GPNMB, DPP4, ERAP2, CTSC, and NPC2) were successfully confirmed. Their levels were generally higher in PTC cystic fluid samples compared to benign cystic fluid samples when equal amounts of total protein were analyzed (Figure 5B). We examined SFTPB as an additional candidate, as it was detected solely in our PTC cystic fluid data set with a very high spectral count (Supplementary Table 4), and was previously reported to be upregulated in PTC tissues [38]. Consistent with these observations, we found that SFTPB was drastically increased in two of the PTC cystic fluid samples (Figure 5A). Taken together, these results indicate that the proteins selected based on our integrated analysis of the FNA cystic fluid proteome, PTC cell secretome, and tissue transcriptome represent strong potential biomarkers for detecting PTC from FNA cystic fluid.

### Overexpression of AGRN, CTSC, ERAP2, and GPNMB in PTC tissues

**Figure 6:**
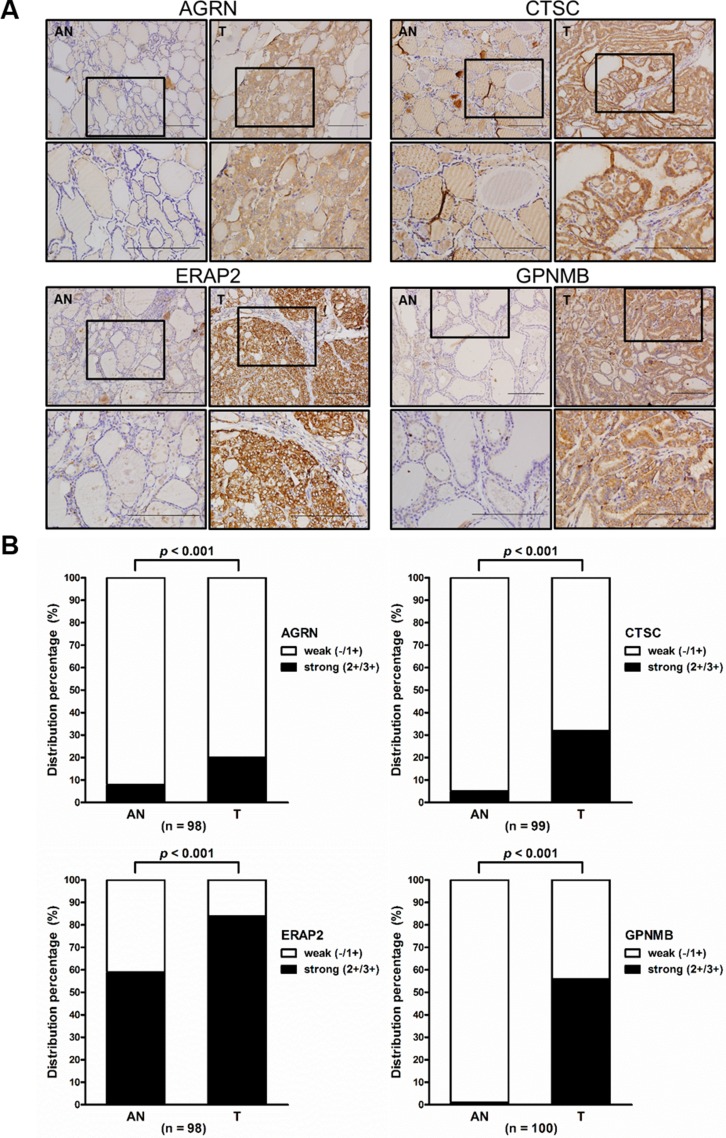

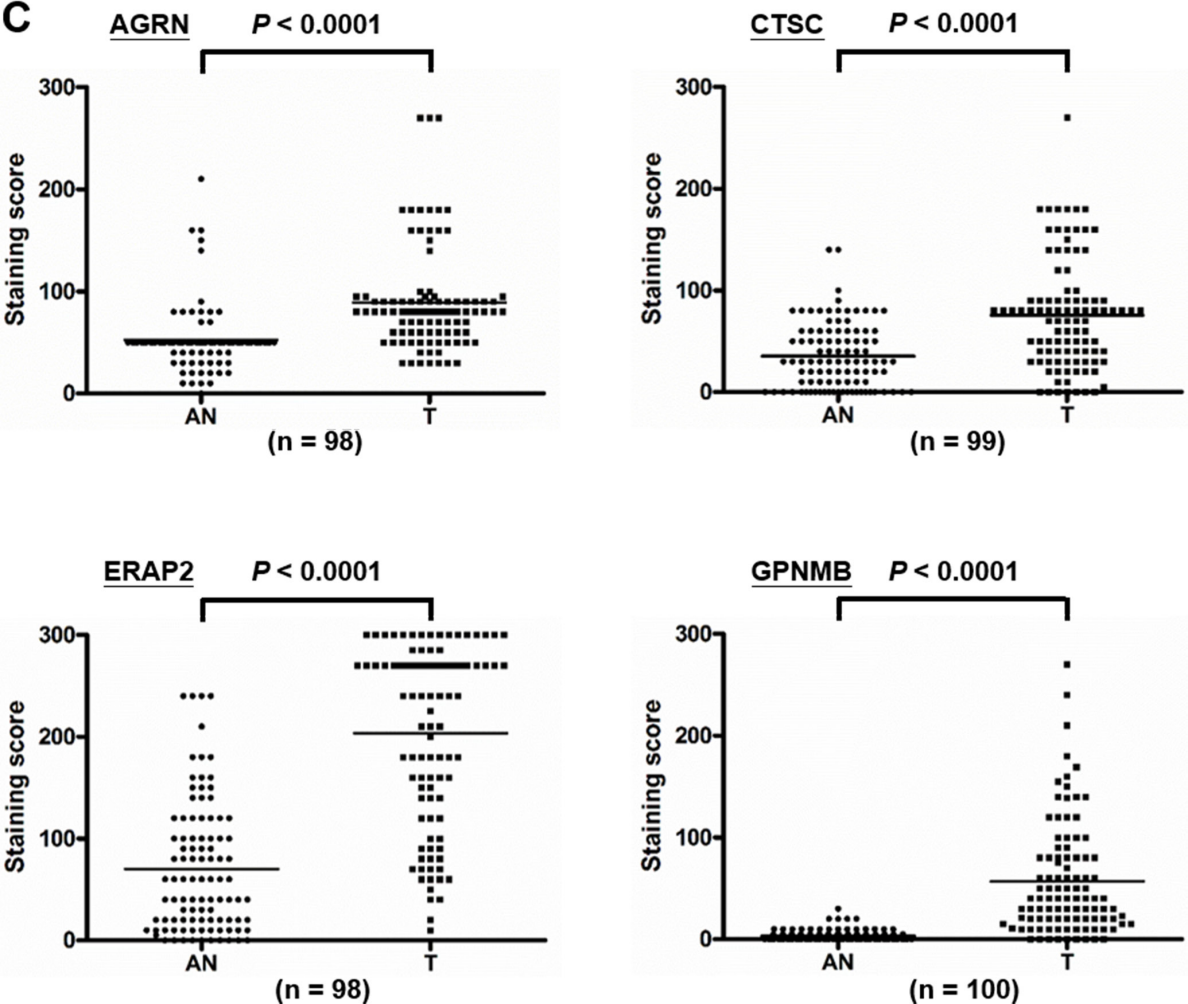
Elevated expression of AGRN, CTSC, ERAP2, and GPNMB in PTC tissues (**A**) Immunohistochemical staining for AGRN, CTSC, ERAP2, and GPNMB in paired tumor (*T*) and adjacent non-tumor (*AN*) tissues from two representative cases (*scale bar* = 200 mm). The boxed areas indicated in the upper panels are enlarged and shown in the lower panels. (**B**) Statistical analysis of the expression levels of AGRN, CTSC, ERAP2, and GPNMB in PTC specimens harboring both tumor and adjacent non-tumor cells. High-level expression is indicated by strong staining (*2+/3+*), whereas low-level expression is indicated by weakly positive (*1+*) and absent (-) staining. (**C**) Statistical analysis of the staining scores from specimens harboring both *T* and *AN* tissues. The dots indicate each case in the scatter dot plot; the middle line indicates the median.

To further examine the expression levels of the seven prioritized targets in PTC tissues, we surveyed antibodies suitable for their immunohistochemical (IHC) analysis. We obtained suitable antibodies against AGRN, CTSC, ERAP2, and GPNMB and used them to stain tissue specimens from 114, 117, 115, and 115 patients, respectively (Supplementary Table 8). Among them, 98, 99, 98, and 100 specimens, respectively, contained PTC tumor and adjacent non-tumor tissues. Representative paired-tissue staining patterns for the four target proteins are shown in Figure 6A. Analysis of the staining scores revealed that strong staining (2+ and 3+) for AGRN, CTSC, ERAP2, and GPNMB was respectively detected in 20.4% (20/98), 32.3% (32/99), 83.7% (82/98), and 56% (56/100) of regions containing tumor cells, while weak staining (1+) was respectively detected in 91.8% (90/98), 94.9% (94/99), 40.8% (40/98), and 99% (99/100) of regions harboring paired adjacent non-tumor cells (Figure 6B). Statistical analysis showed that the expression levels (IHC staining scores) of all four proteins were significantly higher in the tumor parts than in the adjacent non-tumor parts (89.13 ± 49.8 vs. 52.35 ± 30.96, *p* < 0.001 for AGRN; 75.71 ± 54.11 vs. 35.45 ± 32.18, *p* < 0.001 for CTSC; 203.6 ± 88.27 vs. 70.05 ± 63.89, *p* < 0.001 for ERAP2 and 56.78 ± 55.4 vs. 3.3 ± 5.7, *p* < 0.001 for GPNMB) (Figure 6C). It is noteworthy that little or no expression of GPNMB was detected in adjacent non-tumor tissues.

### Associations of AGRN, CTSC, ERAP2, and GPNMB expression with clinicopathological characteristics

We next used the median IHC staining score value as the cutoff for each protein, and explored the relevance of the observed protein expression levels to different clinicopathological characteristics using these IHC stained specimens (114 for AGRN, 117 for CTSC, 115 for ERAP2 and 115 for GPNMB) (Table 4). Higher expression levels of AGRN and CTSC were found to be significantly correlated with lymph node metastasis, distant metastasis at diagnosis, tumor multicentricity, TNM stage, and disease-specific mortality. Notably, high CTSC expression was also significantly associated with an age greater than 45 years and with locoregional recurrence. In contrast, the expression levels of ERAP2 and GPNMB did not show any significant correlation with the tested manifestations.

**Table 4:** Correlations between AGRN, CTSC, ERAP2, and GPNMB expression levels and clinicopathological characteristics of PTC patients

	AGRN			CTSC			ERAP2			GPNMB		
Cut off value: Median	Low (*n* ***=*** 45)	High (*n* ***=*** 69)	*p*-value	Low (*n* ***=*** 57)	High (*n* ***=*** 60)	*p*-value	Low (*n* ***=*** 57)	High (*n* ***=*** 58)	*p*-value	Low (*n* ***=*** 50)	High (*n* ***=*** 65)	*p*-value
Gender^a^, *n* (%)												
Female	33 (73.3)	46 (66.7)	0.535	43 (75.4)	40 (66.7)	0.316	39 (68.4)	42 (72.4)	0.686	36 (72)	46 (70.8)	1
Male	12 (26.7)	23 (33.3)		14 (24.6)	20 (33.3)		18 (31.6)	16 (27.6)		14 (28)	19 (29.2)	
Age (years)^a^, *n* (%)												
<45	24 (53.3)	25 (36.2)	0.084	35 (61.4)	17 (28.3)	0.0004^c^	30 (52.6)	21 (36.2)	0.092	25 (50)	25 (38.5)	0.257
≥45	21 (46.7)	44 (63.8)		22 (38.6)	43 (71.7)		27 (47.4)	37 (63.8)		25 (50)	40 (61.5)	
Tumor size (cm)^a^, *n* (%)												
<2	9 (20)	23 (33.3)	0.259	16 (28.1)	18 (30)	0.374	16 (28.1)	17 (29.3)	0.468	14 (28)	19 (29.2)	0.369
≧2 and ≦4	27 (60)	32 (46.4)		33 (57.9)	28 (46.7)		27 (47.4)	32 (55.2)		24 (48)	37 (56.9)	
>4	9 (20)	14 (20.3)		8 (14)	14 (23.3)		14 (24.6)	9 (15.5)		12 (24)	9 (13.8)	
Lymph node metastasis^a^, n (%)												
No	28 (62.2)	56 (81.2)	0.031^c^	37 (64.9)	50 (83.3)	0.033^c^	41 (71.9)	44 (75.9)	0.675	36 (72.0)	49 (75.4)	0.831
Yes	17 (37.8)	13 (18.8)		20 (35.1)	10 (16.7)		16 (28.1)	14 (24.1)		14 (28.0)	16 (24.6)	
Extrathyroid invasion^a^, *n* (%)												
No	32 (71.1)	50 (72.5)	1	42 (73.7)	41 (68.3)	0.548	43 (75.4)	38 (65.5)	0.308	41 (82)	42 (64.6)	0.058
Yes	13 (28.9)	19 (27.5)		15 (26.3)	19 (31.7)		14 (24.6)	20 (34.5)		9 (18)	23 (35.4)	
Distant metastasis at diagnosis^a^, *n* (%)												
No	43 (95.6)	46 (66.7)	0.0002^c^	54 (94.7)	39 (65)	0.0001^c^	45 (78.9)	47 (81)	0.819	35 (70)	55 (84.6)	0.071
Yes	2 (4.4)	23 (33.3)		3 (5.3)	21 (35)		12 (21.1)	11 (19)		15 (30)	10 (15.4)	
Tumor multicentricity^a^, *n* (%)												
No	36 (80)	41 (59.4)	0.025^c^	44 (77.2)	35 (58.3)	0.032^c^	42 (73.7)	37 (63.8)	0.316	37 (74)	41 (63.1)	0.234
Yes	9 (20)	28 (40.6)		13 (22.8)	25 (41.7)		15 (26.3)	21 (36.2)		13 (26)	24 (36.9)	
TNM stage^a^, *n* (%)												
Stage I-II	29 (64.4)	29 (42)	0.022^c^	40 (70.2)	21 (35)	0.0002^c^	34 (59.6)	26 (44.8)	0.137	28 (56)	31 (47.7)	0.453
Stage III-IV	16 (35.6)	40 (58)		17 (29.8)	39 (65)		23 (40.4)	32 (55.2)		22 (44)	34 (52.3)	
Postoperative 131I cumulative dose (mCi)^b^	232.7 ± 246.5 (1023, 30)	368.4 ± 480.7 (2000, 0)	0.5853	238.9 ± 228.2 (930.6, 30)	389.1 ± 505 (2000, 0)	0.9152	285.5 ± 413.7 (2000, 0)	340.2 ± 381.4 (1370, 0)	0.3215	328.1 ± 483.8 (2000, 0)	304.7 ± 341.4 (1640, 30)	0.2768
Locoregional recurrence^a^, *n* (%)												
No	31 (68.9)	34 (49.3)	0.053	40 (70.2)	27 (45)	0.009^c^	31 (54.4)	35 (60.3)	0.574	25 (50)	41 (63.1)	0.186
Yes	14 (31.1)	35 (50.7)		17 (29.8)	33 (55)		26 (45.6)	23 (39.7)		25 (50)	24 (36.9)	
Disease-specific mortality (%)^a^	4 (9.1)	18 (26.5)	0.029^c^	4 (7.3)	19 (31.7)	0.001^c^	11 (19.6)	11 (19.3)	1	11 (22.4)	11 (17.2)	0.632

Low, IHC score below to the median. High, IHC score equal to or above the median. The p-values were generated using ^a^Chi-square test, number (%) or ^b^Mann-Whitney test, mean ± SD (maximum, minimum). ^c^Statistically significant, *p*-value ≦ 0.05.

### Association of AGRN and CTSC expression with disease-specific survival (DSS)

**Figure 7:**
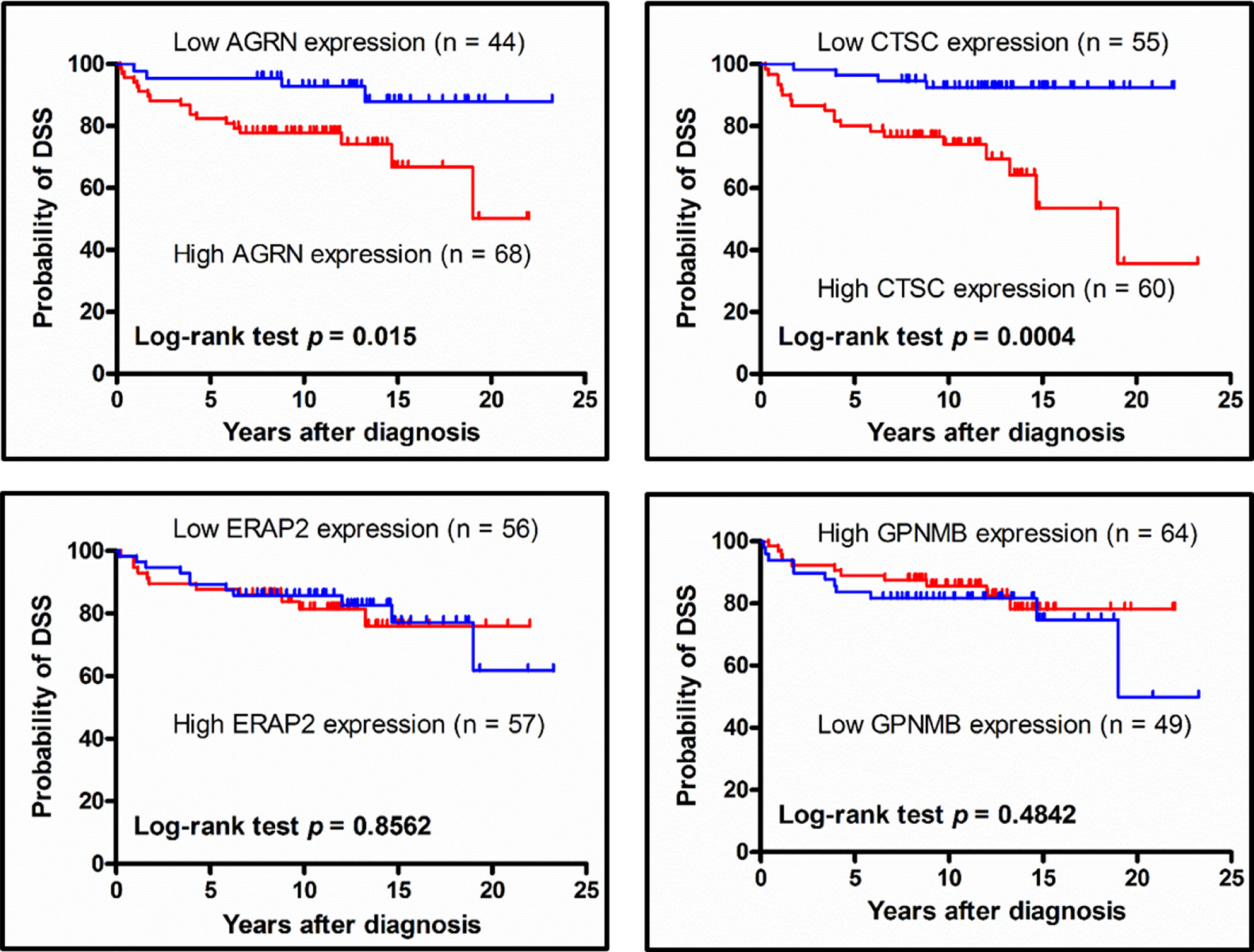
Association between disease-specific survival of PTC patients and target protein expression levels, as analyzed using Kaplan-Meier plots PTC patient subgroups were stratified by high- and low-level expression of AGRN, CTSC, ERAP2, or GPNMB, and then analyzed for their disease-specific survival using a Kaplan-Meier plot. The log-rank test *p*-value is denoted in each plot. Two patients died of other causes but not thyroid cancer had been excluded from this analysis. Thus, the numbers of patients used for this analysis are 112, 115, 113 and 113 for AGRN, CTSC, ERAP2 and GPNMB, respectively.

To assess the correlation the expression of select proteins and patient survival, we used Kaplan-Meier plots to estimate the DSS rates of PTC patients. We found that the 5-year DSS for patients stratified by low or high protein expression were respectively 95.5% vs. 80.9% for AGRN (*p* = 0.015) and 96.4% versus 78.3% for CTSC (*p* = 0.0004), whereas there was no significant expression-related difference in DSS for ERAP2 or GPNMB (Figure 7). These results indicate that the tissue expression levels of AGRN and CTSC appear to be correlated with the DSS of PTC patients.

## DISCUSSION

The integrated analysis of multiple omic datasets has shown promise for the identification of potential biomarkers and/or therapeutic targets for different cancer types [39, 40]. Here, we set out to discover novel PTC biomarkers that are overexpressed in PTC tumors and can be secreted/released by PTC cells, and thus may be detected at elevated protein levels in PTC cystic fluid samples. Through an integrated analysis of in-house-generated FNA cystic fluid proteome and cell line secretome datasets, as well as public-domain tissue transcriptome datasets, we identified seven proteins that fit the above criteria for an ideal PTC biomarker (Figure 3). Further immunoassays confirmed that the levels of four or five of the seven target proteins appeared to be elevated in PTC cystic fluids or tumor tissues (Figures 5 and 6). Higher tumor tissue expressions of two proteins, AGRN and CTSC, were found to be significantly associated with lymph node metastasis, distant metastasis at diagnosis, and poor prognosis of PTC patients (Table 4). Our findings demonstrate that an integrated analysis of multiple omic datasets can be used to identify PTC biomarker candidates that may be of high clinical utility.

Several groups have previously applied proteomic approaches to discover potential PTC biomarkers [8, 10]. The study performed by Martínez-Aguilar et al. is of particular interest. The authors used SWATH-MS (sequential windowed acquisition of all theoretical fragment ion mass spectra) and MRM-HR (high-resolution multiple reaction monitoring) to analyze the proteomes in frozen thyroid tissues that included normal, follicular adenoma, follicular thyroid carcinoma, and PTC samples. They identified 1512 proteins in PTC tissues; of them, ∼180 proteins were deregulated in PTC tumors compared to normal tissues [12]. When we compared the PTC tissue proteome dataset (1512 proteins) with our present cystic fluid proteome dataset (537 proteins), we found 303 proteins commonly detected in both datasets (Supplementary Table 9). Among these 303 proteins, 101 proteins (33.3%, 101/303) showed similar up- or down-regulation trend in both datasets; 73 were significantly up-regulated (≧2 fold) or solely detected in PTC, and 28 proteins were significantly down-regulated (≧2 fold) in PTC or solely detected in benign lesions. Notably, five out of the seven candidates identified in our present integrated analysis of multiple omic datasets (NPC2, CTSC, GPNMB, ERAP2, and SH3BGRL3) were among the proteins that Martínez-Aguilar et al. identified as being up-regulated (>1.5 to 4 fold increase) in PTC versus normal tissues; however, the levels of AGRN were down-regulated and DPP4 was not detected in PTC tissue specimens (Supplementary Table 9). Different approaches and study materials used in our current study and the work from Martinez-Aguilar et al. may account for the discrepancy of proteomic findings between the two datasets. Consistent with the previously reported SWATH-MS data made by Martínez-Aguilar et al., our IHC analysis of ∼100 tissue specimens also revealed that CTSC, GPNMB, and ERAP2 are overexpressed in PTC (Figure 6). Indeed, these three proteins have been consistently found to be overexpressed in PTC samples analyzed by different technologies in distinct areas of the world, including transcriptome analysis of specimens from Finland [32], SWATH-MS analysis of specimens from Australia [12], and our IHC analysis of specimens from Taiwan (this study). Our integrated analysis added further evidence that these proteins could be good PTC biomarker candidates by showing that they could be secreted/released by PTC cell lines and detected at elevated levels in FNA cystic fluids (Figures 4 and 5).

Moreover, we also included a quantitative cystic fluid proteome dataset analyzed by Dinets et al. [11] for further comparison. The authors used iTRAQ labeling coupled with LC-MS/MS approach to analyze the proteome in high abundant proteins-depleted cystic fluid samples from 7 benign and 6 PTC patients. They identified 1581 and quantified 841 proteins in cystic fluid samples. This integrated analysis (191 PTC-specific proteins from our cystic fluid proteome, 2105 proteins from our cell secretome and 1834 proteins from both tissue proteome of Martinez-Alguilar’s study and cystic fluid proteome of Dinets’s study) identified 82 proteins commonly detected in our and Martinez-Alguilar’s (or Dinets’s) datasets (Supplementary Figure 3 and Supplementary Table 10). Among them, 41 proteins with more than two-fold up-regulation (PTC/N) and 23 proteins with down-regulation in PTC lesions were observed in the tissue dataset of Martinez-Alguilar et al. study. Regarding the Dinets’s dataset, 19 up-regulated and 39 down-regulated proteins could be identified, but most of them had fold-change (PTC/Benign) ≧2 (Supplementary Table 10). Four out of seven candidates we identified (NPC2, DPP4, SH3BGRL3 and GPNMB) were also detected in Dinets’s dataset, with up-regulation of NPC2 (1.7 fold) and DPP4 (1.5 fold), down-regulation of GPNMB (0.35 fold) and no obvious change of SH3BGRL3 (1.07 fold) in PTC cystic fluid. As expected, both similarity and differences in the proteomic findings could be found in our and other datasets. Nevertheless, the strategy to integrate different PTC-related tissue and cystic fluid proteome datasets represents a useful mean to prioritize more potential PTC markers for follow-up.

Although several potential PTC biomarkers have been identified in the present study, there are several limitations of our approach. First, only a single proteomics analysis of a single pooled cystic fluid of each clinical group and secretome data of only two cancer cell lines were used for the discovery proteomics experiments. This may not be able to capture tumor heterogeneity and thus other potential biomarkers reflecting the tumor heterogeneity. Second, the poor correlation between expression levels of mRNA and protein may complicate the selection of secreted protein candidates deserved for further study. Third, the number of cystic fluid samples used for verification was small. Further study using larger numbers of samples is needed to prove the clinical utility of these candidate biomarkers.

Agrin (AGRN) is a multifunctional heparan sulfate proteoglycan of the extracellular matrix. It is localized in the basement membrane of the vessels and ducts, and may critically regulate blood-brain barrier conformation and/or synaptogenesis at neuromuscular junctions [41]. Significant overexpression of AGRN was observed during neoangiogenesis in liver cirrhosis and hepatocellular carcinoma, supporting the notion that AGRN stimulates tumor vascularization [42]. AGRN can be detected in ascitic fluid from patients with ovarian cancer, and is secreted by small cell lung cancer cells [43, 44]. An immunoscreening of the extracellular proteome of colorectal cancer cells identified AGRN as an antigen that may be recognized by autoantibodies that exist in sera from colorectal cancer patients [45]. These observations together with our data suggest that AGRN, a proteoglycan that can be secreted by cancer cells via the exosomal pathway, may be a promising PTC marker candidate in cystic fluid.

Cathepsin C (dipeptidyl peptidase I, CTSC) belongs to the papain family of proteinases and participates in the catalytic activation of lysosomal cysteine hydrolase and leukocyte-derived serine proteases [46, 47]. CTSC appears to regulate the degradation of extracellular matrix components that is associated with the metastasis of oral and ovarian cancer cells [48–50]. These observations are consistent with our findings that higher expression of CTSC is correlated with a higher percentage of distant metastasis at diagnosis, locoregional recurrence, and poor prognosis of PTC patients (Table 4 and Figure 7).

Glycoprotein nonmetastatic melanoma protein B (GPNMB) is a type 1 transmembrane glycoprotein that contains three binding motifs of heparin sulfate proteoglycan, lysosome and integrin; it is highly expressed in bone, where it modulates osteoblast maturation and matrix mineralization [51, 52]. Overexpression of GPNMB has been correlated with tumor formation and metastasis in melanomas, gliomas, hepatocellular carcinoma, and breast cancer [53–56]. The antibody-drug conjugate, glembatumumab vedotin, in which a fully human monoclonal antibody against GPNMB is linked to the potent cytotoxin, monomethyl auristatin E, has been approved by the Food and Drug Administration for phase II clinical trials in stage III or IV melanoma and GPNMB-expressing metastatic triple negative breast cancer [57, 58]. Although our data do not suggest that GPNMB is a prognostic indicator for PTC, its levels were found to be dramatically elevated in PTC cells and cystic fluid samples (Figures 4 and 5), suggesting that GPNMB is a strong potential candidate for the targeted therapy and/or diagnosis of PTC.

The endoplasmic reticulum aminopeptidases (ERAPs), which include EARP1 and ERAP2 (also called LRAP), play central roles in trimming longer precursors in the endoplasmic reticulum to generate antigenic peptides that are presented on major histocompatibility complex class I (MHC I) molecules [59]. Animal model studies have shown that altered levels of ERAP1 and ERAP2 can facilitate tumor immune evasion [60, 61]. A recent study investigated the expression of both aminopeptidases in a variety of solid tumors and their normal counterpart tissues, and found that the tumor tissues retained, lost, or acquired expression of either or both aminopeptidases, compared to their normal counterparts, depending on the tumor histotype [62]. Of the thyroid specimens examined in this study, only four exhibited high-level expression of both aminopeptidases in tumor cells but none in their normal counterpart tissues. However, our IHC analysis of ∼100 PTC tissue specimens demonstrated that ERAP2 is overexpressed in PTC (Figure 6). Future studies are warranted to clarify the role of ERAP2 in PTC carcinogenesis and its potential as a PTC biomarker.

In conclusion, our data showed the significant overexpression of AGRN, CTSC, ERAP2 and NPC2 in PTC tissues, and the tissue expression levels of AGRN and CTSC were significantly associated with metastasis and poor prognosis of PTC patients. Therefore, we considered that integration of multiple omics profiling datasets, from FNA cystic fluid proteome, cancer cell secretome to tissue transcriptome, can be a useful approach to discover novel PTC biomarker candidates while there were not enough clinical samples for large scale and comprehensive analysis. Further studies such as using workable ELISAs to verify these candidates in cystic fluids from a large cohort of patients are warranted to evaluate their utilities in clinical settings.

## MATERIALS AND METHODS

### Patient characteristics and clinical specimens

The collection and preparation of thyroid cyst fluids were performed as previously described [63]. Briefly, a real-time ultrasonographic machine with a 10-MHz transducer (ALOKA, Tokyo, Japan) was used to detect thyroid nodule and guide fine-needle aspiration with 22- or 25-gauge needles (Becton Dickinson, Singapore). After the fluid was smeared onto slide, air dried and stained by the Romanowsky-based Liu method [64], fine-needle aspiration cytology (FNAC) was performed and the cytological result was interpreted by a pathologist. Before sample collection, the informed consent forms had been acquired from all patients. Based on cytological and pathological examination, we collected 12 FNA cystic fluid samples from Chang Gung Memorial Hospital (Linkou, Taiwan): seven from patients with benign lesions (2 males and 5 females, mean (±SD) age 41.14 ± 13.16 years, age range 21∼55) and five from PTC patients (2 males and 3 females, mean age 47 ± 16.46 years, age range 24∼70). The cytology tests for the PTC patients were positive for cancer. The features of the FNA cystic fluid samples were assessed by histological analysis. Formalin-fixed and paraffin-embedded samples of thyroid tissues were stained with hematoxylin and eosin (H&E). The guidelines found in “Pathology and Genetics of Tumours of Endocrine Organs” (edited by the World Health Organization, 2004) were used to classify the histopathologic features of the tumor specimens, and clinical staging was based on the definitions of the American Joint Committee on Cancer, 2002 [65, 66]. The characteristics of all study subjects are summarized in Supplemental Table 1. This study was approved by the Institutional Review Board of Chang Gung Memorial Hospital (IRB number 99-3565B).

### Depletion of high-abundance proteins from FNA cystic fluid samples

The typical volumes of cyst fluids used for the top 14 abundant protein depletion were 4.28 μl (protein concentration: 233.64 μg/ml) and 8.82 μl (protein concentration: 113.38 mg/ml) which were respectively pooled from 3 malignant and 7 benign cystic fluid samples and then were subjected to depletion of 14 highly abundant proteins using Agilent Human 14 Multiple Affinity Removal System (MARS) columns (4.6 X 100 mm; Agilent, Palo Alto, CA, USA), which harbor antibodies raised against human albumin, IgG, antitrypsin, IgA, transferrin, haptoglobin, fibrinogen, alpha2-macroglobulin, alpha1-acid glycoprotein, IgM, apolipoprotein A1, apolipoprotein A11, complement C3, and transthyretin. Briefly, the FNA cystic fluid sample was prepared at 1 mg/40 µL in ddH_2_O and diluted four-fold (to 1 mg/160 µL) with 120 µL buffer A of the MARS column system. The diluted sample was processed using the suggested column run cycle which was coupled with AKTApurifier 10 fast protein liquid chromatography (FPLC) (GE Healthcare Life Sciences, Piscataway, NJ, USA) including sample loading, flow-through collection (depleted fraction), washing, eluting the bound proteins with buffer B of the MARS column system and re-equilibrating the column for the next run. The depleted and bound fractions were desalted and concentrated with Amicon Ultra-15 Centrifugal Filter Devices (MW cutoff, 3000 Da; Millipore, Billerica, MA, USA). The proteins were then suspended in ddH_2_O, quantified using a Pierce BCA Protein Assay Kit (Thermo Scientific, Hudson, NH, USA), and stored at -80^o^C for further study.

### Cell culture and collection of conditioned media and cells extracts

The PTC cell lines, CGTH W3 and BHP 7-13 were provided from Dr. Jen-Der Lin (Chang Gung Memorial Hospital at Taoyuan, Taiwan) and cultured in RPMI 1640 supplemented with 10% fetal bovine serum and 100 units/mL of penicillin/streptomycin (Invitrogen, Carlsbad, CA, USA) in a humidified 5% CO_2_ atmosphere at 37°C. Cells were grown to approximately 80% confluence in 15-mm culture dishes, washed twice with phosphate-buffered saline (PBS) and once with serum-free medium, and then incubated in serum-free medium at 37°C for 24 h. The conditioned media were harvested and centrifuged at 1000 rpm for 10 minutes to eliminate the suspended cells. A proteinase inhibitor cocktail was added to the supernatants (final concentrations: 1 mM phenylmethylsulfonyl fluoride [PMSF], 1 mM benzamidine, 0.5 μg/ml leupeptin), which were then concentrated and desalted in Amicon Ultra-15 tubes. The cells that had adhered to the dishes were washed twice with PBS and lysed in homogenization buffer (10 mM Tri-HCl, 1 mM EDTA, 1 mM EGTA, 50 mM NaCl, 50 mM NaF, 20 mM Na_4_P_2_O_7_, 1 mM Na_3_VO_4_, 1 mM PMSF, 1 mM benzamidine, 0.5 μg/ml leupeptin, and 1% Triton-X100, pH 7.4) on ice for 15 minutes. The cell lysate was collected, sonicated on ice, and centrifuged at 11,000 rpm for 20 minutes at 4°C. The supernatant was harvested as the cell extract. The BCA protein assay reagent (Thermo Scientific Pierce, Rockford, IL, USA) was used to determine the protein concentrations of the cell extracts and conditioned media, which were then stored at -80°C for further use.

### One-dimensional SDS-PAGE and in-gel tryptic digestion

The abundant-protein-depleted thyroid FNA samples (60 μg each) or the conditioned media of the PTC cell lines (50 μg each) were separated by 8–14% large-gradient SDS-PAGE (gel dimensions: 0.15 × 12.5 × 14 cm) and stained with Coomassie Brilliant Blue. The gel-separated proteins were processed for MS analysis using in-gel tryptic digestion, as previously described [39]. Briefly, each gel lane was cut into 60 (for FNA cystic fluid samples) or 40 (for conditioned media of PTC cells) pieces, destained three times (15 min each time) with 30 mM ammonium bicarbonate containing 40% acetonitrile, dehydrated in 100% acetonitrile, and dried in a laminar flow hood. The proteins were then reduced with 25 mM ammonium bicarbonatecontaining 10 mM dithiothreitol (Merck KGaA, Darmstadt, Germany) at 60°C for 1 h and alkylated with 55 mM iodoacetamide (GE Healthcare Life Sciences) at room temperature in the dark for 30 min. Sequencing-grade modified porcine trypsin (20 μg/ml in 25 mM ammonium bicarbonate; Promega, Fitchburg, WI, USA) was used for protein digestion at 37°C for 18 h, and the tryptic peptides were extracted with 100% acetonitrile containing 1% trifluoroacetic acid. Finally, the peptides were concentrated and dried by lyophilization.

### Reverse phase liquid chromatography-tandem mass spectrometry

For LC-MS/MS analysis, peptide samples were reconstituted in HPLC buffer A (0.1% formic acid), loaded across a reversed-phase trapping column (Zorbax 300SB-C18, 0.3 x 5 mm; Agilent Technologies, Wilmington, DE, USA) at a flow rate of 0.2 μl/min in buffer A, and separated on a 10-cm analytical C_18_ column (inner diameter, 75 μm; New Objective, Woburn, MA, USA) using a 15-μm tip (New Objective). The peptides were eluted using a linear gradient of 0–10% HPLC buffer B (99.9% acetonitrile containing 0.1% formic acid) for 3 min, 10–30% buffer B for 35 min, 30–35% buffer B for 4 min, 35–50% buffer B for 1 min, 50–95% buffer B for 1 min, and 95% buffer B for 8 min, all at a flow rate of 0.25 μl/min. The LC device was on-line coupled with a two-dimensional linear ion-trap mass spectrometer LTQ-Orbitrap (Thermo Fisher, San Jose, CA, USA) operated with the Xcalibur 2.0.7 software (Thermo Fisher). The MS full-scan was set to the 350-2000 Da range and the intact peptides were detected at a resolution of 30,000. The ion signal of (Si(CH_3_)_2_O)6H+ at *m/z* 445.120025 was used as a lock mass for internal calibration. The utilized data-dependent analytical mode alternated between one full-scan MS and six MS/MS scans for the six most abundant precursor ions in the MS survey scan. The *m/z* values selected for MS/MS were dynamically excluded for 40 s. The voltage of the electrospray ionization was 1.8 kV. The MS and MS/MS spectra were both obtained using one microscan with maximum full times of 1000 and 100 ms for MS and MS/MS, respectively. Automatic gain control was performed to prevent the ion trap from becoming overfilled; 5 × 10^3^ ions were collected in the ion trap for the generation of MS/MS spectra.

### MS data analysis and label-free spectral quantification

The RAW files of the spectra obtained from the LTQ-Orbitrap were searched against 20,367 Homo sapiens entries in the SwissProt-human_56.0 database, with trypsin assumed as the digestive enzyme. The MASCOT Daemon algorithm (version 2.2.03; Matrix Science, London, UK) was used for data processing, and one missed cleavage was allowed. The MS tolerance for the monoisotopic peptide window was set to 10 ppm, and the MS/MS tolerance was set to 0.5 Da. Carbamidomethyl cysteines (+57 Da) and oxidation of methionine residues (+16 Da) was set as variable modification. The charge states of the peptides were set to +2 and +3. All DAT files produced by MASCOT Daemon were combined using the Scaffold software (version 2.06.00; Proteome Software Inc., Portland, OR, USA) to evaluate the MS/MS-based peptide and protein identifications. The probabilistic threshold of protein identification was set at ≧95% and the peptide probability was set at ≧95%. The confidence of protein identification was based on the assignment of at least two identified unique peptides. The false discovery rate (FDR) was calculated from comparison of the spectra assigned to a decoy database that the spectra (random database) versus those assigned to a normal database. The decoy database was generated by Mascot of the same size (i.e., number of amino acids) and also the same number of proteins as the original normal database [67]. The GeLC-MS/MS label-free spectral counts were applied to determine protein ratios and further compare protein expression levels, using the previously described algorithm [68, 69]. Briefly, to use the spectral report from the scaffold to quantify protein expression, the spectra of each protein were normalized by all spectra detected, and the normalized values from the malignant and benign parts were expressed as a ratio. The mass spectrometry proteomics data have been deposited to the ProteomeXchange Consortium (http://proteomecentral.proteomexchange.org) via the PRIDE [70] partner repository with the dataset identifier PXD007532.

### Bioinformatic analysis

The proteins identified from the conditioned media of PTC cell lines and FNA cystic fluid samples were analyzed using the SignalP 4.0 and SecretomeP 2.0 servers to predict the secretory pathways used for proteins with or without signal peptides, respectively [29, 30]. The TMHMM 2.0 program was used to predict transmembrane proteins that may have secretory potential [31]. The DAVID Bioinformatics Resources v6.8 [71, 72] was applied to functional annotation of proteins selected from the discovery experiments, including the biological process, molecular function and cellular component.

### Meta-analysis of two PTC tissue mRNA microarray datasets

Two public thyroid cancer tissue microarray datasets, E-GEOD-3678 and GDS1665, were obtained from ArrayExpress of the European Bioinformatics Institute (EBI) and the Gene Expression Omnibus (GEO) of the National Center for Biotechnology Information (NCBI) [32]. The tissue samples used for gene expression profiling were obtained from seven and nine independent PTC patients for the E-GEOD-3678 and GDS1665 datasets, respectively. The two-sample *t*-test was utilized to determine genes whose expression levels were significantly different between PTC and paired normal tissues (*p*-value ≧0.05). The mean intensity of each gene probe was measured from healthy and cancerous groups, the tumor/normal (T/N) ratio was calculated, the ratios were ranked, and the top 5% up- and down-regulated genes were selected. To unify the ID names with those used in the FNA cystic fluid proteome and cell line secretome datasets, the selected gene probe IDs were converted to Swiss-Prot IDs, and comparison was used to select the candidates that most consistently showed high-level expression in cancerous tissues and FNA cystic fluid.

### Western blot analysis

Cell extracts and conditioned media (50 μg protein) were resolved by SDS-PAGE, transferred to PVDF membranes (pore size, 0.45 μm; Millipore), and probed with antibodies against agrin (AGRN) (1:1000 dilution; sc-6166, Santa Cruz Biotechnology, Santa Cruz, CA, USA), CD26/DPP4 (1:1000 dilution; BAF1180, R&D Systems, Minneapolis, MN, USA), NPC2 (1:1000 dilution, sc-166321, Santa Cruz Biotechnology), GPNMB (1:500 dilution; BAF2550, R&D Systems), galectin-3 (LGALS3; 1:1000 dilution; MAB4033, Chemicon International, Temecula, CA, USA), ERAP2 (1:1000 dilution; AF3830, R&D Systems), CTSC (1:1000 dilution, sc-74590, Santa Cruz Biotechnology), SFTPB (PSPB; 1:1000 dilution, sc-133143, Santa Cruz Biotechnology), tubulin (1:3000 dilution; sc-8035, Santa Cruz Biotechnology), and actin (1:5000 dilution; MAB1501, Millipore). The blots were washed three times with TTBS (20 mM Tris-HCl, pH 7.4, 0.5 M NaCl, and 0.05% Tween 20) and incubated with the appropriate secondary antibody (1:3000 dilution of alkaline phosphatase-conjugated anti-rabbit, -mouse or -goat IgG antibodies; Santa Cruz Biotechnology) for 1 h at room temperature. The blots were then washed three times with TTBS and incubated with the CDP-StarWestern Blot Chemiluminescence Reagent (PerkinElmer, Boston, MA, USA) to detect the proteins of interest. For re-blotting, the membranes were stripped with 2% SDS, 100 mM 2-mercaptoethanol and 62.5 mM Tris at 56°C for 45 min with occasional agitation, washed three times with TTBS, and then re-blotted using other antibodies.

### Immunohistochemical analysis

Formalin-fixed and paraffin-embedded tissue specimens from 121 PTC patients were obtained from Chang Gung Memorial Hospital and sliced into 4-μm-thick sections for immunohistochemical (IHC) staining, which was performed using an automatic IHC staining system according to the manufacturer's instructions (Bond; Vision BioSystems, USA). The antibodies used for IHC staining included those against AGRN (1:50 dilution, sc-25528, Santa Cruz Biotechnology), CTSC (1:40 dilution, sc-13986, Santa Cruz Biotechnology), ERAP2 (1:30 dilution, AF3830, R&D Systems), and GPNMB (1:50 dilution, BAF2550, R&D Systems). The IHC staining intensity and percentage in each section were evaluated by an experienced pathologist. Intensity scores of 0, 1, 2, and 3 indicated negative, weak, moderate, and strong staining, respectively, and the percentage score ranged from 0 (0%) to 100 (100%). The two scores were multiplied to obtain the IHC staining score (0 to 300).

### Statistical analysis

The Wilcoxon signed-rank test was used to compare the differences in the IHC staining scores of the target proteins (AGRN, CTSC, ERAP2, and GPNMB) between paired tissue specimens. The percentage of strong (2+/3+) or weak (1+) IHC staining intensity was calculated with McNemar’s test. The Chi-square test was used to estimate the correlation between the expression level of the target protein in the tumor part of the tissue sample with various clinicopathological parameters. The Kaplan-Meier method was used for the disease-specific survival (DSS) analysis, and the differences in DSS were assessed by Log-rank test. For all statistical analyses, a two-tailed *p*-value ≧0.05 was considered statistically significant. Calculations were performed using the SPSS 20.0 software (SPSS Inc., Chicago, IL, USA) and the presented diagrams were generated using GraphPad Prism 5.0 (GraphPad Software, Inc., San Diego, CA, USA).

## SUPPLEMENTARY MATERIALS FIGURES AND TABLES



















## Abbreviations

EBI: European Bioinformatics Institute
ELISA: enzyme-linked immunosorbent assay
FDR: false discovery rate
FNAC: fine-needle aspiration cytology
FPLC: fast protein liquid chromatography
GeLC-MS/MS: in–gel tryptic digestion coupled with liquid chromatography-tandem mass spectrometry
GEO: Gene Expression Omnibus
MARS: multiple affinity removal system
IHC: mmunohistochemistry
iTRAQ: isobaric tags for relative and absolute quantitation
LC-MS/MS: liquid chromatography-tandem mass spectrometry
LTQ-Orbitrap,: linear ion-trap mass spectrometer
MALDI-TOF-MS: matrix assisted laser desorption / ionization-time of flight mass spectrometer
MRM-HR: high-resolution multiple reaction monitoring
NCBI: National Center for Biotechnology Information
PTC: papillary thyroid carcinoma
SWATH-MS: sequential windowed acquisition of all theoretical fragment ion mass spectra
TM: transmembrane
USG/FNA: ultrasound-guided fine-needle aspiration
