# Supplementary material for: Integrated analysis of fine-needle-aspiration cystic fluid proteome, cancer cell secretome, and public transcriptome datasets for papillary thyroid cancer biomarker discovery
Source: Oncotarget. 2018 Jan 4;9(15):12079–100. doi: 10.18632/oncotarget.23951 (PMC5844730; doi:10.18632/oncotarget.23951)
Supplement: Supplementary file 1 [file oncotarget-09-12079-s001.pdf]

Integrated analysis of fine-needle-aspiration cystic fluid proteome, cancer cell secretome, and public transcriptome datasets for papillary thyroid cancer biomarker discovery

SUPPLEMENTARY MATERIALS

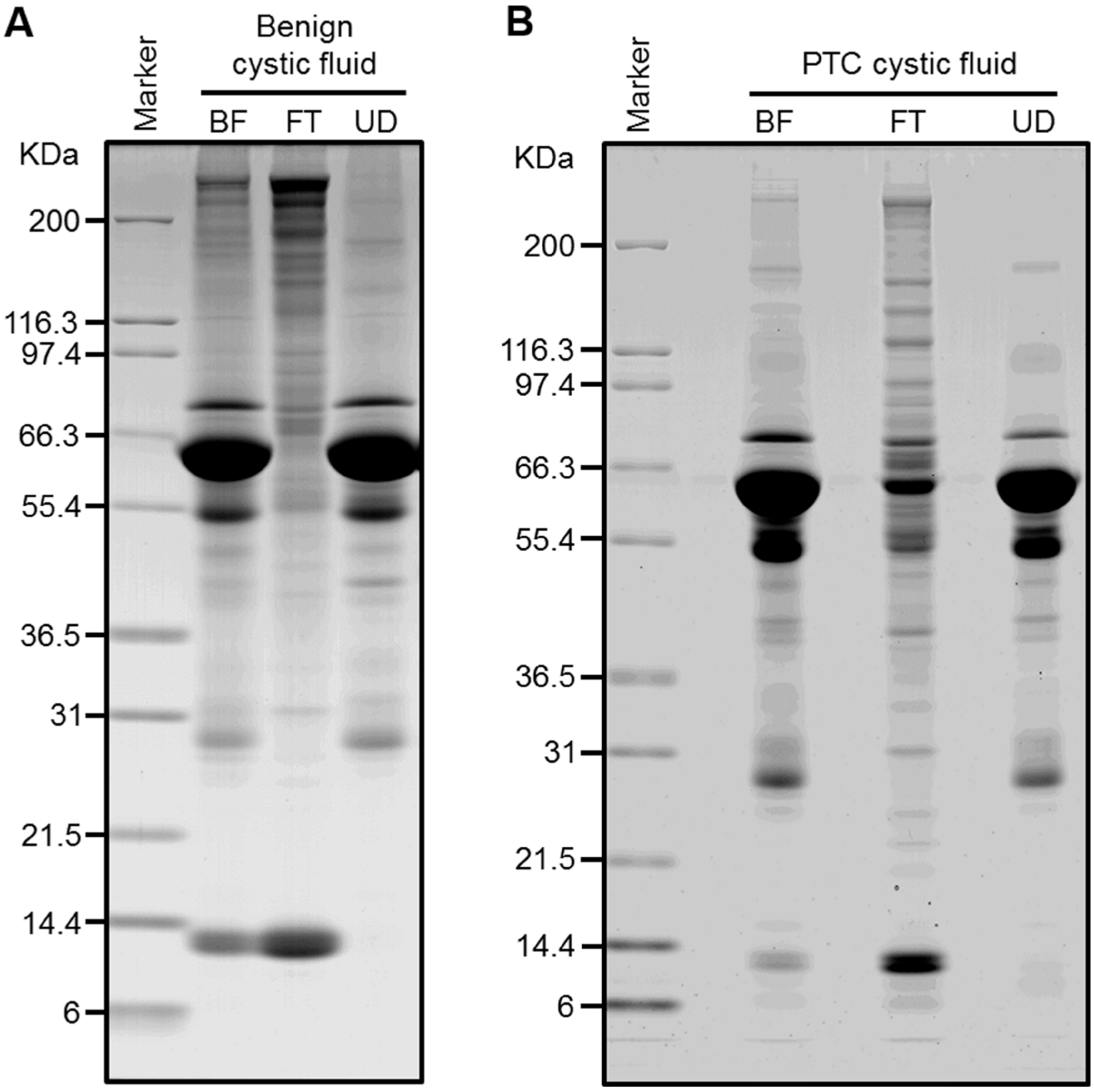

Supplementary Figure 1: SDS-PAGE analysis of thyroid cystic fluids. Thyroid cystic fluids of seven benign samples and three PTC samples were independently pooled and depleted of abundant proteins using Hu-14 columns. Aliquots (60 ug protein) from the binding fraction (BF), flow-through fraction (FT), and original undepleted fraction (UD) were resolved on 8–14%-gradient gels and stained with Coomassie Blue. (**A**) Benign cystic fluid sample. (**B**) PTC cystic fluid sample.

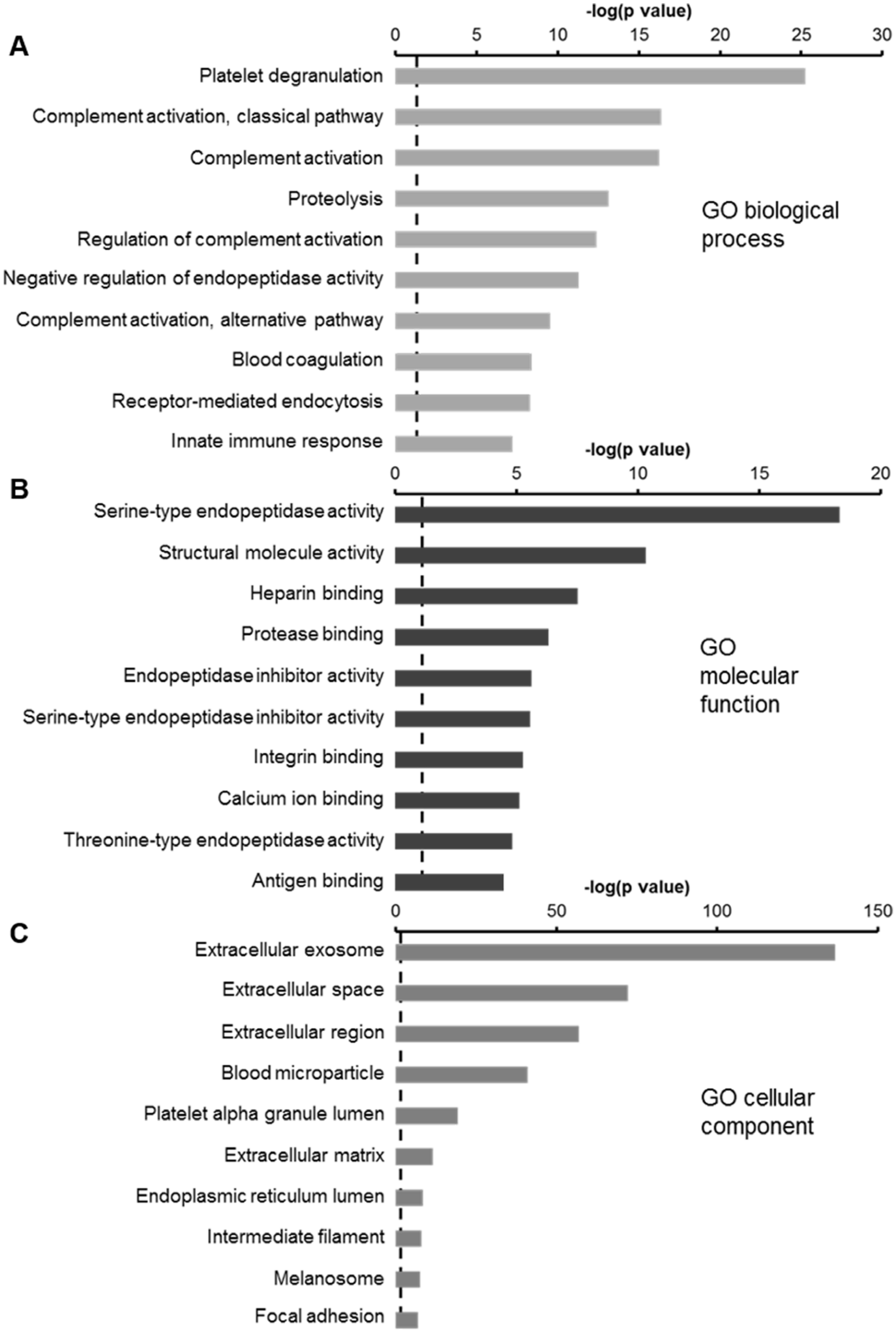

Supplementary Figure 2: Functional classification of those 277 proteins with two-fold up-regulation or solely detected in PTC cystic fluids using DAVID software. Shown here are the top 10 categories in each group: (**A**) biological process, (**B**) molecular function, and (**C**) cellular component. The *dotted line* indicates $p = 0.05$; hence, *columns* to the right of this line are statistically significant.

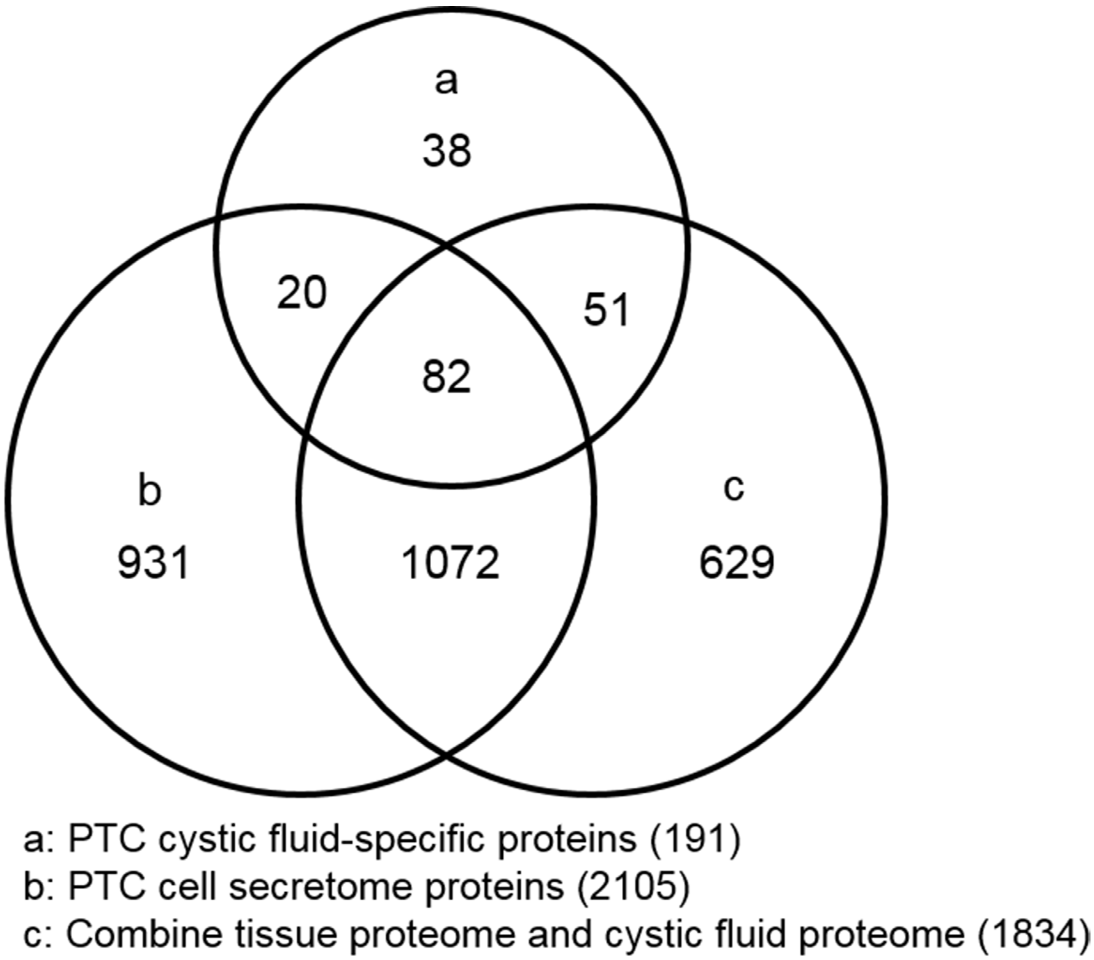

Supplementary Figure 3: Integrated analysis of PTC-related proteome datasets. Venn diagrams showing the overlap of proteins in 191 PTC-specific proteins from our cystic fluid proteome, 2105 proteins from our cell secretome and 1834 proteins from tissue proteome of Martinez-Alguilar's study (Proteomics of thyroid tumours provides new insights into their molecular composition and changes associated with malignancy. Martínez-Aguilar J, Clifton-Bligh R, Molloy MP, PLoS One. 2015 May 15; 10(5):e0126472) and cystic fluid proteome of Dinets's study (Differential Protein Expression Profiles of Cyst Fluid from Papillary Thyroid Carcinoma and Benign Thyroid Lesions. Andrii Dinets, Maria Pernemalm, Hanna Kjellin, Vitalijs Sviatoha, Anastasios Sofiadis, C. Christofer Juhlin, Jan Zedenius, Catharina Larsson, Janne Lehtiö, Anders Höög, Sci Rep. 2016 Mar 30; 6:23660).

Supplementary Table 1: Clinicopathological characteristics of the patients from whom thyroid cystic fluid samples were obtained and studied

Sample No.	Histopatological diagnosis	Sex (M / F)	Age (years)	Tumor size (cm)	TNM Stage
1*	Bening	F	53		
2*	Bening	M	29		
3*	Adenoma	F	34		
4*	Bening	F	50		
5*	Bening	F	55		
6*	Bening	F	46		
7*	Simple Cyst.	M	21		
1*	PTC	M	46	2.9	II
2*	PTC	F	24	6.0	I
3*	PTC	M	70	2.1	II
4	PTC	F	44	3.4	I
5	PTC	F	51	6.0	III

*The samples were used in GeLC-MS/MS analysis. Numbers 1-7 were pooled as the benign sample, while numbers 1-3 of PTC were pooled as the PTC sample.
All 12 samples were subjected to Western blot analysis for candidate marker validation.

Supplementary Table 2A: List of proteins identified from the Hu-14-column-binding proteins during the depletion of benign thyroid cystic fluid. (FDR = 0.6%).

Supplementary Table 2B: List of proteins identified in the undepleted PTC cystic fluid. (FDR = 0.8%).

Supplementary Table 2C: Comparison of proteins identified in the crude (undepleted) and abundant plasma protein-depleted PTC cystic fluids. See_Supplementary_Table 2

Supplementary Table 3A: List of proteins identified from benign thyroid cystic fluid. (FDR = 0.58%)
Supplementary Table 3B: List of proteins identified from PTC thyroid cystic fluid. (FDR = 1.23%)
Supplementary Table 3C: List of proteins identified in conditioned media from CGTH W3 cell line. (FDR = 0.12%)
Supplementary table 3D: List of proteins identified in conditioned media from BHP 7-13 cell line. (FDR = 0.21%) See_Supplementary_Table 3

Supplementary Table 4: List of proteins combined from PTC and benign cystic fluids. See_ Supplementary_Table 4

Supplementary Table 5A: List of proteins identified in conditioned media combined from CGTH W3 and BHP 7-13 cell lines.
Supplementary Table 5B: The seven candidate proteins identified herein, along with LGALS3. See_Supplementary_Table 5

Supplementary Table 6: Numbers obtained from the label-free quantitation of the proteins in thyroid cystic fluids

PTC/Benign cystic fluid protein level	Ratio ≧ 2	2 > Ratio ≧ 0.5	Ratio < 0.5	No. of unique proteins in PTC cystic fluid[a]	No. of unique proteins in benign cystic fluid[b]
Numbers	86	175	36	191	49

The numbers of identified protein spectra were normalized by all spectra (protein spectra/total spectra), and a ratio was calculated between PTC and benign cystic fluid proteins (PTC/benign).
[a]Proteins were solely detected in the PTC cystic fluid dataset.
[b]Proteins were solely detected in the benign cystic fluid dataset.

Supplementary Table 7: PTC tissue microarray datasets. See_Supplementary_Table 7

Supplementary Table 8: Clinicpathological characteristics of the 121 tissue samples used for the immunohistochemical analysis of four candidate proteins. See_Supplementary_Table 8

Supplementary Table 9: Comparative analysis of proteomic datasets obtained from our current cystic fluid proteome (537 proteins) and the tissue proteome of Martínez-Aguilar et al. (1512 proteins). See_Supplementary_Table 9

Supplementary Table 10: Comparative analysis of proteomic datasets obtained from 191 PTC-specific proteins from our current cystic fluid proteome, 2105 proteins from our current cell secretome, tissue proteome from Martinez-Alguilar's study and cystic fluid proteome from Dinets's study. See_Supplementary_Table 10
